# Outstanding Questions and Future Research on Magnetic Reconnection

**DOI:** 10.1007/s11214-025-01143-z

**Published:** 2025-02-11

**Authors:** R. Nakamura, J. L. Burch, J. Birn, L.-J. Chen, D. B. Graham, F. Guo, K.-J. Hwang, H. Ji, Y. V. Khotyaintsev, Y.-H. Liu, M. Oka, D. Payne, M. I. Sitnov, M. Swisdak, S. Zenitani, J. F. Drake, S. A. Fuselier, K. J. Genestreti, D. J. Gershman, H. Hasegawa, M. Hoshino, C. Norgren, M. A. Shay, J. R. Shuster, J. E. Stawarz

**Affiliations:** 1 Space Research Institute, Austrian Academy of Sciences, Schmiedlstraße 6, 8042 Graz, Austria; 2 International Space Science Institute, Bern, Switzerland; 3 Southwest Research Institute, San Antonio, TX 78238 USA; 4 Center for Space Plasma Physics, Space Science Institute, Boulder, CO 80301 USA; 5 Goddard Space Flight Center, NASA, Greenbelt, MD 20771 USA; 6 Swedish Institute of Space Physics, Uppsala, Sweden; 7 Los Alamos National Laboratory, Los Alamos, NM 87545 USA; 8 Department of Astrophysical Sciences, Princeton University, Princeton, NJ 08544 USA; 9 Department of Physics and Astronomy, Dartmouth College, Hanover, NH 03750 USA; 10 Space Science Laboratory, UC Berkeley, Berkeley, CA 94720 USA; 11 University of Maryland, College Park, MD 20742 USA; 12 The Johns Hopkins University Applied Physics Laboratory, Laurel, MD 20723 USA; 13 The University of Texas at San Antonio, San Antonio, TX 78249 USA; 14 Institute of Space and Astronautical Science, JAXA, Sagamihara, Japan; 15 Department of Earth and Planetary Science, The University of Tokyo, Tokyo, 113-0033 Japan; 16 Department of Physics and Astronomy, University of Delaware, Newark, DE 19716 USA; 17 Space Science Center, University of New Hampshire, Durham, NH 03824 USA; 18 Department of Mathematics, Physics, and Electrical Engineering, Northumbria University, Newcastle upon Tyne, UK

**Keywords:** Magnetic reconnection, Magnetospheric Multiscale (MMS) mission, Diffusion region, Onset, Cross-scale, Energetics

## Abstract

This short article highlights unsolved problems of magnetic reconnection in collisionless plasma. Advanced in-situ plasma measurements and simulations have enabled scientists to gain a novel understanding of magnetic reconnection. Nevertheless, outstanding questions remain concerning the complex dynamics and structures in the diffusion region, cross-scale and regional couplings, the onset of magnetic reconnection, and the details of particle energization. We discuss future directions for magnetic reconnection research, including new observations, new simulations, and interdisciplinary approaches.

## Introduction

Magnetic reconnection is a fundamental energy conversion process in plasmas. While changes in the topology of the magnetic field take place inside a small region, acceleration and heating of the plasma are distributed over larger scales. Acceleration and heating drive plasma transport and lead to explosive magnetic energy release likewise on large scales during phenomena such as substorms, solar flares and gamma ray bursts. With modern space technology, geospace is an ideal plasma laboratory for studying how collisionless magnetic reconnection operates in nature since plasmas and fields in action can be directly measured at high cadence. With the advanced in-situ measurement capability to resolve electron-scale physics, the four Magnetospheric Multiscale (MMS) spacecraft (Burch et al. 2016) have significantly advanced the study of magnetic reconnection and relevant plasma processes. The rich studies conducted in the MMS era motivated us to summarize the current understanding of magnetic reconnection that arises from new observations mainly in geospace and in other environments as well as from theoretical studies (Burch and Nakamura 2025, this collection).

Studies based on in-situ observations from MMS and numerical simulations confirmed some theoretical predictions and led to a number of new discoveries on dynamics of reconnection at smallest scale: the electron kinetic scale (Genestreti et al. 2025, this collection). In particular, progress has been made in observations and theories related to the reconnection rate and energy conversion processes (Liu et al. 2025, this collection), and in the kinetic behavior of both electrons and ions in the vicinity of the diffusion region (Norgren et al. 2025, this collection). The diverse roles of waves and turbulence in magnetic reconnection are also among the important discoveries from the MMS observations (Graham et al. 2025, this collection; Stawarz et al. 2024, this collection). Some of these features were not predicted or not the focus of theory or numerical simulations before the MMS era.

MMS, combined with other spacecraft and empirical and/or theoretical modeling, has allowed us to gain new insights into the macroscale consequences of reconnection. These include the large-scale consequences of solar-wind magnetospheric interactions (Fuselier et al. 2024, this collection) and particle acceleration (Oka et al. 2023b, this collection), as well as the coupling among magnetic reconnection-related processes at different scales (Hwang et al. 2023, this collection). All these studies benefited from the development of new data analysis techniques (Hasegawa et al. 2024, this collection) and simulation/modeling schemes (Shay et al. 2025, this collection), which allow direct comparisons between the observed and simulated velocity distributions of particles and electromagnetic signatures.

Recent observations throughout the different environment in the solar system (Drake et al. 2025, this collection; Gershman et al. 2024, this collection) and advanced laboratory experiments (Ji et al. 2023, this collection) enabled us to study different scales of magnetic reconnection in different parameter regimes and deepen our understanding of the process. New kinetic and fluid simulations have also significantly contributed to understanding magnetic reconnection in both collisionless and collisional astrophysical plasmas (Guo et al. 2024, this collection).

While significant advancements in magnetic reconnection research have been made with these endeavors, there remain several unsolved questions. These questions relate to kinetic physics and macroscale consequences in different environments, both within and beyond geospace. In this short paper, we highlight several unsolved questions and propose future research directions in the short term (years) using MMS as well as in the long term (decades).

## Unsolved Problems

### Complex Dynamics and Structures in the Diffusion Region

Substantial progress has been made in understanding the relationship between magnetic reconnection and kinetic plasma waves (e.g., Graham et al. 2025, this collection). These include specification of the types and locations of the waves that can develop during reconnection and identification of particle distributions that can excite the waves (e.g., Burch et al. 2018). However, much less is known about the effects of these waves on the plasma and it is likewise not well known how these waves can affect reconnection. In particular, an ongoing question is whether anomalous resistivity due to wave-particle interactions contributes to magnetic reconnection, for example by modifying the reconnection electric field (e.g., Yoo et al. 2024). MMS was able to directly quantify the anomalous resistivity associated with reconnection by resolving the changes in electron distributions and moments associated with lower hybrid waves around the X-line (Graham et al. 2022). The results revealed that the anomalous resistivity balances with anomalous viscosity so that its contributions to the reconnection electric fields were small, which is consistent with the findings of previous theoretical and observational studies. However, these waves contribute to significant cross-field diffusion that can develop and thereby broaden narrow boundary layers and facilitate electron mixing. Further work can be done with MMS to answer the question on the role of waves in reconnection by examining the interactions between electron and higher-frequency waves. While the current direct investigation of wave-particle interaction using the highest-resolution electron distributions is limited to the lower hybrid frequency range, the wave-particle correlator technique can be applied to reconnection current sheets to study higher-frequency wave-particle interactions. This technique has been used to compute the energy transfer between waves and particles for whistler waves in the magnetosheath (Kitamura et al. 2022).

Furthermore, MMS has produced discoveries that have not been predicted by theory or numerical simulations. MMS observations have shown that the agyrotropic electron distributions found in the electron diffusion region (EDR) can become unstable to large-amplitude waves (Graham et al. 2025, this collection), such as upper hybrid waves and electron Bernstein waves, due to beam-plasma interactions. These waves provide potential sources of radio emission and can modify the electron distributions in the EDR, but their overall impact on reconnection remains to be quantified. These observations also clearly demonstrate the presence of physical processes at scales below the electron gyroscale, i.e. down to the Debye scale, inside the EDR. The proper description of EDR physics must therefore include Debye-scale processes, which are often only marginally resolved in typical simulations (see Sect. 3.3).

MMS observations have also shown that some EDRs exhibit turbulent structures (Khotyaintsev et al. 2020) or strong oscillations (Cozzani et al. 2021) in and around EDRs. The oscillations were attributed to kinking of the current sheet by an electromagnetic drift wave propagating in the out-of-plane direction, suggesting that magnetic reconnection needs to be considered in three dimensions. Kinetic simulations have shown that EDRs can become structured and turbulent when there is scale separation between the electron Debye length and the electron inertial length (Jara-Almonte et al. 2014). More generally, MMS observations have reported both turbulent and more laminar EDRs at the magnetopause and in the magnetotail (Liu et al. 2025, this collection; Graham et al. 2025, this collection). At present, the underlying processes that determine whether an EDR behaves in a laminar or turbulent manner are not fully understood. This raises the important question of whether more complicated EDRs are missed or overlooked in observations. Although many EDRs have been identified by MMS, their identification has generally relied on predictions from kinetic simulations of laminar reconnection. Further work is needed to identify more complex EDRs. Methods such as tunable algorithms (e.g., Bergstedt et al. 2020) or machine-learning techniques (e.g., Argall et al. 2020; Hasegawa et al. 2024, this collection; Bergstedt and Ji 2024) can be applied to identify relevant magnetic structures from observations. With more EDRs, case studies, which have dominated the research so far, can give way to statistical studies. This transition leads to a more comprehensive understanding of complex EDR dynamics.

At present, guide-field reconnection is not as well understood as antiparallel reconnection. Electrons in the EDR tend to remain strongly magnetized in the presence of a strong guid field. When the electrons are magnetized, the off-diagonal pressure terms play a reduced role in supporting the reconnection electric field. This is in contrast to antiparallel reconnection when the reconnection electric field is supported by the off-diagonal pressure terms generated by electron agyrotropy, which is often used to identify EDRs. Kinetic simulations demonstrate the formation of a narrow sublayer (of intensified current density) embedded within the broader, electron inertia-scale EDR (Liu et al. 2014b). The off-diagonal pressure term only becomes significant within this sublayer, which is on the electron gyro-scale (Genestreti et al. 2025, this collection). Additionally, a strong guide field creates out-of-plane field-aligned electron flow around the X-line. This electron flow is free energy for the development of electrostatic waves and turbulence in the EDR. The reduced role of agyrotropy and the role of electrostatic turbulence in guide-field reconnection requires further investigation. Interestingly, the same out-of-plane electron flow from magnetic reconnection in the strong guide field limit may explain some features of electron precipitation associated with the auroral spiral structure (Huang et al. 2022).

### Cross-Scale Dynamics and Regional Coupling

**Fig. 1:**
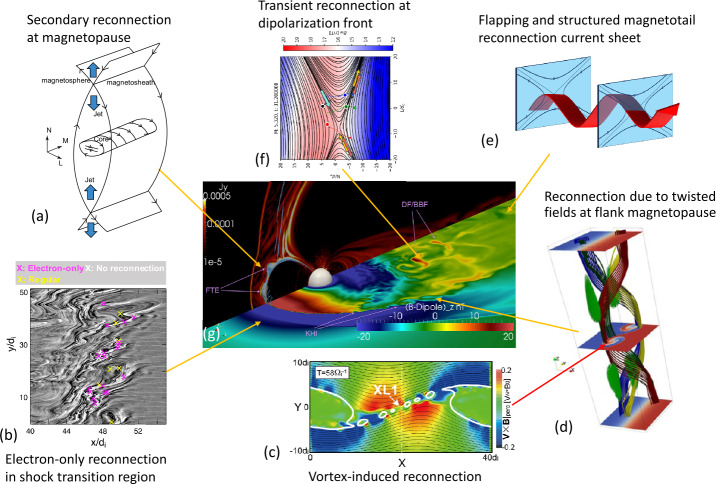
**Reconnection in geospace**. In addition to global dayside and nightside magnetic reconnection, recent in-situ measurements and simulations have revealed 3D, complex, and localized reconnection features throughout geospace. (a-f) Examples of different types of reconnection that are actively studied in the MMS era. (g) 3D view of the magnetosphere from an MHD model (Credit: V. G. Merkin) adapted from Sitnov et al. (2016), where several key mesoscale processes KHI, BBF/DF and FTE related to localized reconnections are indicated. The highlighted reconnection features are: (a) secondary and/or multiple reconnection at the magnetopause (adapted from Øieroset et al. 2016), (b) turbulent reconnection in the shock transition region (adapted from Bessho et al. 2022), (c-d) 3D multiscale KHI/KHV induced reconnection (adapted from (c) Nakamura et al. 2011 and (d) Faganello et al. 2012), (e) structured and disturbed EDR in the magnetotail current sheet (adapted from Cozzani et al. 2021) and (f) transient and localized reconnection at the dipolarization front (adapted from Hosner et al. 2024)

Magnetic reconnection operates in the presence of a diffusion region with dissipative electric fields which are generated in the EDR. Electron-kinetic physics prevails in the EDR, whereas Hall physics becomes significant in the ion diffusion region (IDR). The influence of magnetic reconnection further extends to macroscopic systems, such as magnetospheric boundaries and mesoscale plasma structures in geospace, for which ideal magnetohydrodynamics (MHD) provides a good overall description. These nested reconnection regions around the X-line are interconnected via the exchange and transport of particles, momentum, energy and Poynting flux. Thus, reconnection is intrinsically a multiscale and cross-scale process all the way up to the macroscale. In-situ observations and state-of-the-art numerical simulations have significantly advanced our understanding of the multiscale aspects of reconnection (Hwang et al. 2023, this collection) occurring throughout geospace, as highlighted in Fig. 1. They also revealed new questions, as discussed in the following sub-sections. By answering these questions, they may change the current understanding, leading to a paradigm shift.

#### Electron-Only to Ion-Coupled Reconnection

MMS data-model analyses have shown that reconnection is ubiquitous in the shock transition region, the foreshock, and the magnetosheath downstream of both quasi-parallel and quasi-perpendicular shocks (Fig. 1b, adapted from Bessho et al. 2022). Of particular interest in this region is the electron-only reconnection, newly discovered in observations (Phan et al. 2018), which has stimulated new theoretical studies (Liu et al. 2025, this collection) and new investigations on the interplay between turbulence and reconnection (Stawarz et al. 2024, this collection). In turbulent systems, electron-only reconnection is considered to occur mainly because the scale of the turbulent fluctuations limits the maximum size of the reconnection region, particular along the reconnection outflow. Alternatively, it has been suggested that electron-only reconnection might represent the early stage of regular reconnection before the reconnection exhaust becomes large enough to involve ions (e.g. Hubbert et al. 2022). Such finite lifetime effects may be relevant for magnetotail reconnection. However, confirming such a scenario is challenging. In the simulations, electron-only reconnection was shown to have faster reconnection rate than for regular reconnection (Sharma Pyakurel et al. 2019). It is uncertain observationally whether the transition from electron-only to ion-coupled reconnection is regulated by the reduction in the reconnection rate. Further investigations and observations are needed to gain a complete understanding of electron-only reconnection, its role in cross-scale reconnection dynamics, and the scale-dependent energy conversion.

#### Velocity Shear-Driven Asymmetric Reconnection

Asymmetries in density and magnetic shear are important factors in different regimes of reconnection (Genestreti et al. 2025, this collection). These effects as well as the effects of flow shear are prominent at the flank-side magnetopause. In this region, flow shear drives the complex multiscale evolution of the magnetopause current sheet, which can develop into a turbulent layer depending on the ambient conditions (Hwang et al. 2023, this collection; Stawarz et al. 2024, this collection). When the interplanetary field (IMF) is northward, the unperturbed low-latitude magnetopause on the flank is stable to the formation of extended reconnection diffusion regions, but unstable to the large-scale Kelvin-Helmholtz instability (KHI) driven by shear flows. Under super-Alfvénic conditions the vortex flow produced by the nonlinear growth of the KHI can locally compress the magnetic shear layer (current sheet), forcing the onset of vortex-induced reconnection (VIR) (Nakamura et al. 2017). Multiple reconnection regions appear in the current sheet as shown in Fig. 1c and can result in a complex turbulent boundary layer. When the IMF is southward, meaning a strong magnetic shear at the magnetopause favorable for reconnection, the evolution of the current sheet varies depending on the initial condition (magnetic shear vs. flow shear). However, the two modes can interact with each other, leading to complex and intercorrelated dynamics. Understanding the interplay between reconnection and the KHI (and/or Rayleigh-Taylor instability associated with density asymmetry) is important, as it would control solar wind transport and energy conversion across the flankside magnetopause. Furthermore, reconnection can also occur around the flow-shear plane due to a 3-D twist of the magnetospheric and magnetosheath magnetic fields induced by Kelvin-Helmholtz vortices (Faganello et al. 2012). This type of reconnection is shown in Fig. 1d and is called “mid-latitude reconnection” (MIR). MIR occurs several Earth radii apart from the low-latitude VIR location, while being magnetically connected in 3D. Hence, the potential “communication” between the two reconnection sites can affect solar wind transport in a complex way. These examples show that magnetic reconnection at the flank-magnetopause provides an excellent laboratory for studying multiscale (forced) 3D reconnection.

#### Extent and Orientation of X-Lines; Primary and Secondary X-Lines

While magnetic reconnection at the magnetopause and magnetotail is considered the driver of global magnetosphere circulation, reconnection in these large-scale currents has variability in space and time and signatures of multiple reconnection sites (Fuselier et al. 2024, this collection; Hwang et al. 2023, this collection). Interpreting in-situ reconnection events is often complicated as the large-scale context of reconnection cannot be ascertained from observations with limited coverage. There remain unsolved questions regarding the temporal and spatial scales of reconnection in mesoscale and large-scale contexts for both the magnetopause and the magnetotail.

At the magnetopause, the location and extent of the primary X-line are considered to be determined by the global solar wind-magnetosphere interaction, enabling us to predict this interaction via the maximum magnetic shear model (Trattner et al. 2007; Hasegawa et al. 2024, this collection, and reference therein). This is an empirical model that uses upstream conditions and global parameters. However, observations have also revealed more complicated structures, with localized and transient behavior of multiple reconnection sites at the magnetopause. An example is magnetic reconnection at the center of a magnetic flux rope where the jets from the adjacent two reconnection sites collide and form a compressed thin current sheet (Fig. 1a, adapted from Øieroset et al. 2016). Some simulations suggest that local physics can influence the orientation and variation of the X-line (e.g., Liu et al. 2018). The relationships between the primary and secondary X-lines are yet unsolved problems. Are secondary reconnection sites created by turbulence or external (e.g., magnetosheath) conditions near the primary X-line? Alternatively, is the migration of the primary X-line initiated by the local physics of the secondary reconnection sites? Lastly, is it possible that multiple X-lines develop simultaneously, with the roles of primary vs. secondary being later established? The evolutionary paths of plasmoids and flux ropes commonly generated on the dayside magnetopause via secondary/multiple X-lines are also yet to be understood.

The configuration of the magnetotail current sheet is typically quasi-2D and symmetric, so that the formation of a large-scale extended X-line is expected; however, the statistical distribution of observed reconnection events suggests that the near-Earth magnetotail reconnection is localized within ∼5 Earth radii (Nagai and Shinohara 2021). One of the major challenges with observations is determining the extent of the reconnection region in the out-of-plane direction (as reviewed in Hwang et al. 2023, this collection). The dawn-dusk extent of bursty bulk flows (BBFs), associated localized dipolarization fronts (DFs) and localized thin current sheets can be more easily detected due to their larger cross-section (relative to the diffusion region). The finite extent of these transients may indicate a finite dimension of the source, i.e., the magnetic reconnection region. Alternatively, their size may be decoupled from that of their source as (a) a ballooning/interchange instability may break up a wider flow into localized channels as it penetrates into the inner magnetosphere or (b) the structured flows/DFs may be created by the interchange instability itself. Furthermore, transient localized reconnection can also take place at a DF (Fig. 1f adapted from Hosner et al. 2024) so that the DF is modified as it propagates Earthward from the source region. Understanding the extent of the reconnection region is crucial, as it affects large-scale dynamics, e.g., magnetic flux and mass transport, as well as particle acceleration in the magnetosphere. Recently, the application of data mining tools has provided some insight into the extents of X-lines (Stephens et al. 2023). The larger spacecraft separations along the MMS spacecraft orbit planned in 2024 may enable new studies of 3D nature of X-lines including the out-of-plane direction in the Earth’s magnetotail. Furthermore, as discussed in Sect. 2.1, MMS reported strong oscillation along the X-line inside EDRs (Fig. 1e adapted from Cozzani et al. 2021). Such complex dynamics in the diffusion region may affect the extent of the X-line and the local reconnection rate (Liu et al. 2024).

### Onset of Reconnection

While the free energy of reconnection is determined by the large-scale background plasma conditions and has large-scale consequences, the dissipation of the tearing mode occurs at the ion or electron gyro-scale/gyroradii. The onset problem is therefore multiscale in nature, and an under-explored topic in reconnection physics. Limited coverage of all necessary scales by in-situ plasma observatories makes it very difficult to compare with theoretical/numerical descriptions. Here we highlight the onset problems of different types of current sheets including magnetotail, solar flares, magnetopause and other forced current sheets.

#### Reconnection Onset in Earth’s Magnetotail

To understand the onset of near-Earth magnetotail reconnection one needs to understand both the formation of the thin current sheet and the instability leading to explosive energy release. The observed thin current sheets are generally embedded in a thicker plasma sheet with anisotropy and agyrotropy in both ions and electrons and contain radial or azimuthal gradients (Runov et al. 2021, and references therein). Detection of the formation and evolution of thin current sheets from in-situ observations is limited because of the sparse dataset. The current best approach to resolve multiscale current sheet structures is the data-mining method (Sitnov et al. 2019b), which helped resolve the location of the X-lines (Stephens et al. 2023) detected by the MMS mission in the form of IDRs.

MHD models suggest that thin current sheets are created because of the deformation of the high-latitude magnetopause boundary by the reconnected and transported magnetic flux from the dayside (Birn and Schindler 2002) or because of depletion of the closed magnetic flux at the near-Earth current sheet transported toward the dayside (Hsieh and Otto 2014). The basic concept of the former effect was obtained in the isotropic plasma description of MHD models and was also verified via 2D PIC simulations (Hesse and Schindler 2001). However, modeling of the onset current sheet with very small, but still finite $B_{Z}$ (normal component to the current sheet), where the anisotropic and agyrotropic pressure contributions play a role, is challenging. The mechanism leading to the onset of magnetotail reconnection with finite $B_{Z}$ has been extensively studied via simulations, which revealed two primary onset scenarios (Sitnov et al. 2019a, and references therein). The first is the electron tearing instability preceded by an external driving of the current sheet as described above to form an electron scale current sheet (e.g. Hesse and Schindler 2001; Liu et al. 2014a). The second is a magnetic flux release instability in an ion-scale current sheet with a $B_{Z}$ hump (Sitnov and Schindler 2010). It may develop in the ideal-MHD regime and in the form of the kinetic ion tearing instability. The problem is, however, that simulations of both the ion and electron tearing instability reveal that the new X-lines form just ∼15 $d_{i}$ (<2$R_{E}$) from the left boundary of the simulation box, which is much closer to Earth than nearly all observed X-lines in the magnetotail. Recently a new class of current sheets has been explored (Sitnov and Arnold 2022). In these current sheets, weak anisotropy in the ion species extends them much farther than corresponding isotropic (Harris-like) current sheets. The new “overstretched ion-scale current sheets” are agyrotropic and are supported by the off-diagonal pressure gradient terms originating from ion Speiser motions (Arnold and Sitnov 2023). However, comprehensive stability theories for these new current sheets have yet to be developed and simulations of reconnection onset are still an active area of research.

Using in-situ observations to detect reconnection onset is another challenge. A recent particle-in-cell (PIC) simulation suggested that a possible observable onset feature is a slightly agyrotropic electron distribution (Spinnangr et al. 2022). However, to date, there is no identified MMS observations within less than 10 ion gyroperiods from onset in the vicinity of the EDR to confirm such predictions. Nevertheless, several observations of MMS electrons near current sheets are suggested to indicate precursors of larger scale reconnection onset and these observations are consistent with predictions from simulations. These include: observations of thin electron-scale current sheets with slow electron flows (Wang et al. 2018), divergent electron velocity flow observations without magnetic topology change (Motoba et al. 2022), and observations of electron-scale islands and Hall currents in the vicinity (or as a consequence of the formation) of a major X-line (Genestreti et al. 2023). However, all these observations are snapshots of some stages of reconnection evolution predicted by some simulations. Multiscale observations, which monitor both the ion- and electron-scale evolution of the current sheet simultaneously, are essential for confirming the different onset mechanisms of fast reconnection in the magnetotail current sheet.

#### Reconnection Onset in Solar Flares

The mechanisms of flare onset and associated particle accelerations are also a research area with outstanding questions (Drake et al. 2025, this collection). Similar to magnetotail reconnection, both the build up and trigger for the sudden release of magnetic energy need to be explained to understand the mechanism of solar flare onset. As in the magnetotail, the large-scale accumulation of energy preceding reconnection onset and its transport down to kinetic length scales are important for solar flares in coronal loops; hence it is again a multi-scale problem. While solar kinetic scales are inaccessible from observations, the complex 3D evolution of solar flares has been extensively studied through multiwavelength observations as well as in-situ measurements of accelerated particles that propagate away from coronal loops. Theories for magnetic reconnection onset in flares, such as breakout (Antiochos et al. 1999) and tether cutting (Jiang et al. 2021), have been successful in producing standard eruptive morphologies such as a twisted CME flux rope escaping at high speed and fast reconnection in the flare current sheet below the flux rope. The kink instability of flux ropes in the solar corona (Török and Kliem 2005), on the other hand, has also been suggested to be important for flare onset. However, it has not been established definitively from observations or simulations whether Alfvénic motions cause the onset and drive reconnection or vice versa (Drake et al. 2025, this collection). Furthermore, observed local precursors such as preflare-heating and its role in subsequent eruption, remain to be understood (e.g. Battaglia et al. 2019; Hudson et al. 2021).

In contrast to the near antiparallel geometry of the magnetotail current sheet, the guide field plays a crucial role in the evolution of the reconnection current sheet in solar flares. In the presence of a strong guide field, the thermal pressure of the current sheet cannot play a major role in the force balance, since the guide field contributes to magnetic pressure at the center of the reversal and prevents the collapse of the converging fields (Leake et al. 2020; Dahlin et al. 2022). It is also possible for a current sheet with a small finite guide field to evolve toward a “mixed” equilibrium, where the current sheet relaxation process leads to local guide field amplification (Yoon et al. 2023). The amplification of the guide field enhances the previously negligible magnetic pressure, and creates a condition where both the thermal pressure and the magnetic pressure play a significant role in stabilizing the current sheet (Yoon et al. 2023). A similar guide field amplification process has been reported in a 3D MHD simulation study (Dahlin et al. 2022). Here, a local accumulation of magnetic shear followed by outward expansion to form a thin current sheet was shown immediately before flare onset, after which the guide field decreased precipitously. Strong magnetic shear has also been associated with larger and more rapid increases in ion kinetic and thermal energy after reconnection onset in the corona, making it a potential candidate to explain the switch-on nature of solar flares (Leake et al. 2020). The role of other instabilities, such as the kink instability, in flare onset is still an open question (Drake et al. 2025, this collection).

The dynamics of reconnection in flare current sheets span an enormous range of scales in a much more complex geometry than in the magnetotail. In a collisional plasma with high Lundquist number ($\sim 10^{14}$) such as the solar corona, the Sweet-Parker current layers are highly unstable to the plasmoid instability (Shibata and Tanuma 2001; Loureiro et al. 2007; Bhattacharjee et al. 2009). These layers are modified well before they can reach kinetic scales and current sheet breakups have been successfully simulated with fluid models (Daldorff et al. 2022). In thin currents that form between flux-ropes, on the other hand, the super-Dreicer fields induce a transition to kinetic reconnection (Stanier et al. 2019), which cannot be detected from observations. How the dynamics of reconnection current layers at kinetic scales couple to energy release at the macroscale is still an open question (Drake et al. 2025, this collection).

#### Reconnection Onset in Different Forced Current Sheets

The onset problems of magnetic reconnection in the magnetotail and solar flares discussed in the previous sections are about a slow build-up of a thin current sheet followed by a sudden onset. Magnetic reconnection can also be forced to take place by external drivings. In this subsection, we discuss how such forced magnetic reconnection takes place. We highlight magnetopause reconnection and reconnection at the other transient forced current sheets. Forced reconnection has been intensively studied in the MMS era. In forced current sheets, the reconnection onset problem is less related to “when” but is more related to “where” and “under what conditions”.

The magnetopause current sheet is continuously driven by the solar wind. Magnetopause reconnection is enabled or disabled depending on the asymmetries in the density and the magnetic and flow shear across the magnetopause current sheet. These factores are reviewed in Hwang et al. (2023, this collection) and Fuselier et al. (2024, this collection) for geospace and in Gershman et al. (2024, this collection) for planetary magnetosphere and heliopause. The diamagnetic drift stabilization (Swisdak et al. 2010) or shear flow-based suppression (Cassak 2011) provide sufficient but not necessary conditions for determining where reconnection is suppressed. The suppression conditions have been successfully tested at Earth and planetary magnetospheres. However, the Earth’s magnetopause does not fulfill typically the diamagnetic-drift stabilization condition, i.e., reconnection is ‘possible’ for the typical range of changes in plasma $\beta $ across the terrestrial magnetopause over large range of magnetic shear angles (Cassak and Fuselier 2016). The mechanism of determining the location of magnetopause reconnection as well as the multiple and transient nature of the magnetopause reconnection is therefore not fully understood (see also Sect. 2.2).

Numerous studies have shown that local, transient thin current sheets form and reconnect as a consequence of reconnection (or non-reconnection) related flows or field disturbances (Hwang et al. 2023, this collection; Stawarz et al. 2024, this collection), as discussed in Sect. 2.2. Examples of such current sheets are highlighted in Fig. 1. Unlike large-scale magnetopause or magnetotail current sheets, these current sheets can be localized and/or transient within complex dynamic processes. These include flow shear (Kelvin-Helmholtz instability) driven reconnection at the flank magnetopause (Nakamura et al. 2017), shown in Fig. 1c, and shock- and turbulent-driven reconnection in the magnetosheath or foreshock region (Bessho et al. 2022), shown in Fig. 1b. Open questions remain on interplay between reconnection and turbulence such as: how often reconnection can be generated by turbulence, how a turbulence-generated current sheet is forced by fluctuation, and what impact magnetic reconnection has on turbulence dissipation and nonlinear interactions (Stawarz et al. 2024, this collection). Furthermore, the reconnection jet itself can also be a driver of secondary reconnection due to the collision of reconnection jets from multiple X-lines (Øieroset et al. 2016), shown in Fig. 1a. In the near-Earth magnetotail transition region, reconnection events have been reported where a flux rope interacted with the dipole field (Poh et al. 2019), or at the dipolarization front in the flow braking region (Marshall et al. 2020; Hosner et al. 2024), shown in Fig. 1f. These types of reconnection events are usually forced by some primary processes. Important questions are: how these primary processes create such current sheets and how these reconnection events subsequently affect the overall system. Exploring different regions in space with dedicated in-situ measurements may lead to the further discoveries of different types of thin current sheets throughout the solar system.

### Energetics, Acceleration, and Heating

The energy explosively released through magnetic reconnection goes into plasma flow energy, heating, and nonthermal particle acceleration in systems ranging from electron-scale current sheets to magnetospheres of accreting black holes. The nature and controlling factors of energetics in the vast array of reconnection systems are among the most compelling questions in reconnection research. Recent developments in laboratory (Ji et al. 2023, this collection), geospatial (Oka et al. 2023b, this collection), solar (Drake et al. 2025, this collection) and astrophysical (Guo et al. 2024, this collection) investigations present an unprecedented opportunity to establish a common framework for energetics across different systems. In the following sections, we list long-standing open questions, and in particular, highlight how the released magnetic energy is partitioned between thermal and nonthermal components and between electrons and ions in the realms of magnetotail observations, solar flares, astrophysical systems, and laboratory experiments.

#### Magnetotail Observations

In-situ observations in the magnetotail enable the study of particle acceleration at various regions related to reconnection, e.g., the diffusion region, separatrix, magnetic islands or flux ropes, outflows and the dipolarization front (Oka et al. 2023b, this collection). Distinct power law spectra for both electrons and protons are associated with reconnection. The partition between the non-thermal and thermal populations varies for different magnetic reconnection events. A puzzle is that a significant increase of the thermal electron population is not always associated with a hard non-thermal tail (Oka et al. 2022). One should note that a nonthermal population is also observed in a quiet plasma sheet. How particles are heated and accelerated to non-thermal energies in the magnetotail remains to be understood. For ions, there are fewer studies on the energy partition between thermal and nonthermal components. A recent study suggests that ion energization is dominated by electric field fluctuations near the ion cyclotron frequency (Ergun et al. 2020). How energies are partitioned between ions and electrons is also an important unsolved problem. When the ion and electron energy fluxes were compared in the ion diffusion region of magnetotail reconnection, they were dominated by the ion enthalpy flux, with smaller contributions from the electron enthalpy flux and the heat flux and the ion kinetic energy flux (Eastwood et al. 2013).

The important role of turbulence in particle acceleration was identified in low-$\beta $ magnetotail reconnection events for both ions and electrons (Ergun et al. 2020). While the formation of the nonthermal tail distribution is generally considered on the basis of the guiding-center approximation, how particles interact with turbulence/waves and how they receive energization “kicks” from fluctuations, which are inherently nonadiabatic interactions, remain open questions. It is also interesting to learn how turbulence regulates the repartitioning of energy released by reconnection as a function of distance from the X-line, since energy may be transferred from the bulk outflow into the particle thermal energy or kinetic energy of energetic particles over some distance. Another factor affecting the energization processes in magnetotail reconnection is the finite extent of the reconnection regions and their multiplicity, as discussed in Sect. 2.2. Electrons and low-energy ions have gyroradii smaller than the typical size of the reconnection outflows and can be confined within the reconnection region. However, heavier or energetic ions can have a gyroradius comparable to the transverse scale of the reconnection outflow and thus can no longer be trapped within the outflow, and their acceleration may stop. For such ions to gain further increase in the energy, they need to interact with multiple reconnection events. However, such structures and the evolution of multiple reconnection sites in the magnetotail are difficult to identify from observations. A further caveat that must also be considered in magnetotail events is that the particle distribution observed from a spacecraft prior to an event is generally not identical to the source population observed afterward. Understanding the energetics of reconnection in the magnetotail via simultaneous coverage of the acceleration regions in a larger context, i.e. from the X-line to the outflow regions, is essential.

#### Solar Flares

Macroscale energy release from magnetic reconnection has been extensively observed through remote sensing of solar flares, and also via recent in-situ measurements of the near-sun solar wind related to interchange reconnection within the coronal holes and reconnection in the heliospheric current sheet (Drake et al. 2025, this collection). Solar flare observations first suggested that the magnetic energy released during reconnection is partitioned into nonthermal and thermal components of electrons and ions. In contrast to magnetotail reconnection, data suggest that the contributions of nonthermal electrons are comparable to or exceed those of thermal electrons. Significant ion energy gains are detected in the emission, although the observed emission is limited in energy range. Combining in-situ observations of flare ejecta from the Parker Solar Probe and Solar Orbiter is expected to improve our understanding of ion energetics.

Theory and modeling efforts have significantly advanced our understanding of the macroscale particle acceleration mechanisms related to reconnection, as summarized in Drake et al. (2025, this collection). Different models that integrate MHD with particle descriptions have been shown to be effective in producing power-law spectra (Arnold et al. 2021; Li et al. 2022; Yin et al. 2024a,b; Seo et al. 2024). These models, as they cover kinetic to large-scale MHD regimes, make it possible to compare and predict imaging spectroscopy observations of solar flares and the highest energy particle acceleration in astrophysical objects. To improve understanding of the energetics of flare reconnection, it is essential to compare observations and model predictions of the role of the guide field or location of the acceleration sites.

#### Astrophysical Systems

In astrophysical systems, magnetic reconnection has been proposed as a mechanism to explain high-energy phenomena and radiation signatures in systems such as pulsar wind nebulae, pulsar magnetospheres, relativistic jets, gamma-ray bursts, accretion disks, and magnetars (Uzdensky 2011; Hoshino and Lyubarsky 2012; Arons 2012; Guo et al. 2020). Magnetic reconnection can take place in relativistic magnetically dominated regions in these systems. High-energy emissions are observed during reconnection. Heating versus acceleration is one of the key issues in the reconnection studies as discussed in the review by Guo et al. (2024, this collection). Relativistic reconnection events trigger acceleration in various regimes where the power-law tail slope can approach unity (Sironi and Spitkovsky 2014; Guo et al. 2014; Werner et al. 2016; Li et al. 2023). Direct acceleration due to the reconnection electric field can also lead to power-law spectra (Zenitani and Hoshino 2001) in addition to the more common Fermi/betatron processes among different systems (Guo et al. 2015, 2019). However, the overall framework of the energy partition problem is similar to those of other systems. Treating the large-scale fluid behavior and the basic particle acceleration process simultaneously is a challenging problem as in other systems, considering the enormous ratio between the system size and the plasma kinetic scales. Different theories have successfully explained magnetic reconnection as a source of nonthermal particles. Many unresolved questions (e.g., how much energy goes to thermal and nonthermal) are also relevant to space plasmas, but the phenomena exist at much more varied scales, including those observed surrounding black holes in the Event Horizon Telescope.

#### Laboratory Reconnection Energetics

With the advantage of being able to systematically quantify reconnection energetics, laboratory experiments have made substantial progress on this topic (Ji et al. 2023, this collection), particularly when combined with numerical simulations and space observations. As the magnetic energy is converted into flows, thermal and nonthermal energization takes place at the X line, separatrices, exhausts, and far downstream. Consistent with space observations and fully kinetic simulations, the ion energy gain exceeded that of the electrons in laboratory experiments of reconnection (Yamada et al. 2018). Recent experiments detected directly accelerated electrons via reconnection electric fields and nonthermal electrons for anti-parallel reconnection in low-$\beta $ plasmas (Chien et al. 2023). These new experiments are expected to enable new comparative studies with space-based reconnection studies. The range of system sizes achievable in laboratory experiments is thus far within 10 ion-inertial lengths from the X-line; hence, the aspects of dynamics and energy conversion at global scales are open challenges. The effects of plasma collisions need to be carefully handled for comparative studies with space plasma. Future experiments in new facilities such as FLARE (Ji et al. 2022) will access both the collisional and collisionless regimes, promising fruitful comparisons with magnetic reconnection in space and astrophysical systems.

## Future Research

The outstanding questions reviewed in the previous section motivate us to advance the current observation and computing capabilities, and to think beyond existing capabilities. In this section, we discuss new research aspects that can increase our understanding of magnetic reconnection in space plasma environments.

### Interdisciplinary Studies

The recent developments in magnetic reconnection in astrophysical systems has strong connections with reconnection in space, solar and laboratory environments and these connections can be extended further in the future. The development of collisionless magnetic reconnection and kinetic simulations, starting in the 1990s in the space plasma community, laid the solid ground for studying relativistic magnetic reconnection in the astrophysics community. It has become common knowledge that kinetic physics supports fast magnetic reconnection and that magnetic reconnection likely leads to plasma heating and particle acceleration (Birn et al. 2012, and references therein). The other way around, the development of relativistic magnetic reconnection led to new knowledge and motivations for reconnection physics and particle acceleration mechanisms applicable to the nonrelativistic regime. For example, recent progress of theories of reconnection rate was initiated by studies of relativistic magnetic reconnection (Liu et al. 2017). The development of nonthermal powerlaws in simulations of relativistic magnetic reconnection removed doubts surrounding the development of such spectral forms in the nonrelativistic case (Guo et al. 2024, this collection). Motivated by these advances, particle power-law distributions have recently been achieved in nonrelativistic studies (Arnold et al. 2021; Li et al. 2019; Zhang et al. 2021, 2024). Such connections and communication between different communities should continue, and discussions should be strongly encouraged.

Through the common framework of theory and simulations, processes occurring in solar and astrophysical systems captured with large-scale remote-sensing images can be bridged to those in space and laboratory environments where plasma is “directly” measured. The understanding and knowledge gained from in-situ kinetic-scale measurements in geospace and in the laboratory can be applied to other planetary environments. This knowledge also serves as a foundation for understanding larger scale systems such as solar flares and astrophysical phenomena (e.g., relativistic jets in quasars). Direct comparison of the energy spectra between solar flare and magnetotail reconnection has proven to be a successful scheme for studying particle acceleration in magnetic reconnection (Oka et al. 2023a; Drake et al. 2025, this collection). The efficiency of reconnection in the solar wind-planetary interaction uses the common framework observed throughout the solar system (Fuselier et al. 2024, this collection; Gershman et al. 2024, this collection) and serves as a reference for other stellar systems. The 3D dynamics and evolution of the reconnection current sheet detected from in-situ measurements (Hwang et al. 2023, this collection) as well as in controlled laboratory settings (Ji et al. 2023, this collection) benefits from the knowledge of larger scale context gained from solar flare studies (Drake et al. 2025, this collection) and vice versa. That is, to identify the energy conversion site and its dynamics in the solar context one can take into account knowledge from in-situ observations such as those made by MMS (Genestreti et al. 2025; Liu et al. 2025; Norgren et al. 2025; Graham et al. 2025; Stawarz et al. 2024, all in this collection). Communications between communities with different skill sets and bases of knowledge are essential.

### Multiscale Observations

As outlined in Sect. 2.2, cross-scale dynamics and regional coupling remain challenging, unsolved problems. Ion-scale and electron-scale physics have been studied by multi-spacecraft missions such as Cluster and MMS, respectively, and THEMIS has enabled studying larger scale evolution. However, it is necessary to have a larger number of spacecraft covering a wide range of scales simultaneously. In order to realize observations for answering the cross-scale science questions, various future mission concepts have been proposed. These include *Plasma Observatory* (Retinò et al. 2022), which would cover simultaneously the ion and fluid scales at different magnetosphere boundaries, and multipoint observations with sufficient energy range to study terrestrial magnetotail reconnection, including the larger context, such as *MagneToRE* (Maruca et al. 2021), *MagCon* (Kepko et al. 2023), *WEDGE* (Turner et al. 2023), and a CubeSat constellation mission *AME* (Dai et al. 2020).

It would also be interesting to study the distant magnetotail, as reconnection signatures have been identified in the far downtail region ($X \sim $ −100 to −200 $R_{E}$). While the future multi-spacecraft mission *HelioSwarm* (Klein et al. 2023), is designed to study solar wind turbulence, and also crosses the magnetotail down to $X \sim $ −60 $R_{E}$, it is important to push further downtail beyond this distance. Such an extension in observational capabilities would allow us to study larger-scale reconnection signatures, including chains of plasmoids, and enable some comparisons with solar flares. These comparions are possible despite the fact that the ion kinetic scale in the magnetotail is on the order of 100–1000 km, whereas it is only 1 m in the solar corona.

Improving solar flare observations is also crucial for facilitating interdisciplinary and comparative studies. In the next few years, the *Solar-C* (Shimizu et al. 2020) and *MUSE* (Cheung et al. 2022) missions will be launched. These missions will study reconnection-related phenomena by conducting spectroscopic observations at EUV wavelengths with wide and seamless temperature coverage (1–1000 eV) and high temporal and spatial resolutions. However, to understand energetics and fast-varying plasma processes such as shocks and reconnection, it is also important to conduct imaging spectroscopy via X-rays (e.g. Oka et al. 2023a; Glesener et al. 2023). Unlike EUV emissions, which can be delayed due to ionization and recombination processes (e.g. Imada et al. 2011; Shen et al. 2013), X-ray continua are produced via bremsstrahlung emission without delay. Recent advancements in photon-counting techniques and improved focusing optics are likely to cover large dynamic ranges at high temporal and spatial resolutions. Therefore, high-precision imaging-spectroscopy of reconnection-related phenomena is expected to be realized. The energy spectrum is obtained seamlessly from thermal to nonthermal energy ranges, which is a crucial step toward a better comparative study between solar and space plasmas. Currently, mission concepts such as *PhoENiX* (Narukage et al. 2020) and *FIERCE* (Shih et al. 2023) are being developed to achieve such imaging spectroscopy via X-rays.

### Future Numerical Simulations and Modeling

Currently, modeling of magnetic reconnection largely relies on numerical simulations, as presented in Shay et al. (2025, this collection). In PIC simulations, various parameters such as the mass ratio ($m_{i} / m_{e}$) and the ratio of the plasma frequency to the electron cyclotron frequency ($\omega _{pe} / \omega _{ce}$), are often not realistic to reduce the computational cost. There is currently no consensus on how realistic these parameters should be to provide physically meaningful results. These parameters need to be chosen carefully since the artificial mass ratio controls the separation between ion-scale and electron-scale physics and modifies plasma wave properties. Interestingly, Debye-scale turbulence was reported to alter electron-scale dynamics (Jara-Almonte et al. 2014) when a realistic frequency-ratio parameter is used.

A major unsolved area of research is the interaction of magnetic reconnection with both mesoscale and global-scale dynamics. By “mesoscale,” we mean that the length scales are much larger than the ion diffusion region but still smaller than the global magnetospheric scales. Examples of such multiscale interactions are the generation and dynamics of bursty bulk flows in the magnetotail as well as the interaction of reconnection and turbulence both in the magnetosheath and upstream of the Earth’s bow shock. A major issue with studying these multiscale interactions is that PIC simulations are too computationally expensive to include meso- and global-scales. To capture the multiscale nature of magnetic reconnection and its interaction with global-scale dynamics, several novel numerical schemes such as interlocking PIC and MHD models (Daldorff et al. 2014; Tóth et al. 2016) have been developed. Additionally, a recently proposed hybrid simulation model, *kglobal*, couples particle gyrokinetics with MHD simulations for particle acceleration studies (Shay et al. 2025, this collection).

Several new directions are emerging, both in software and hardware. Owing to the strong requirements for electric power, recent supercomputers have begun to use “accelerators” such as graphic processing units (GPUs). Since the programming model is different, it is often necessary to develop GPU variants of simulation codes. A growing number of simulation codes have been recently developed for GPUs to overcome the issue of yet-to-be-improved software development environments. Another new direction is machine learning (ML) or artificial intelligence (AI) technologies (Camporeale et al. 2024). ML/AI is useful not only for postprocessing the simulation data but also for predicting solutions for physics problems (Raissi et al. 2019; Karniadakis et al. 2021). Furthermore, quantum computers could be game changers (Grumbling and Horowitz 2019), although the timeline for the creation of practical hardware for simulations is still unknown. They may allow us to calculate a far larger number of variables than classical computers do. However, since basic principles and logic circuits are very different, the development of algorithms for new simuations is required to start from scratch. In the next decade, when algorithms and hardware are further advanced, the role of quantum computing in plasma simulations is expected to become clearer.

## Conclusions

Recent advancements in in-situ plasma measurements, which have enabled the study of collisionless magnetic reconnection physics, including kinetic physics, led to new discoveries as well as many of the open questions discussed in the previous sections. While they address examples mostly from geospace, many of these open questions are also applicable to other systems including other planets, astrophysical systems, and laboratories. However, in-situ measurements are limited by a specific range of observed plasma parameters and specific scales. Remote observations, on the other hand, usually cover the large-scale context of magnetic reconnection but have a limited energy range and limited resolution that does not cover the microscale. Future observational capabilities addressing the multiscale problems of magnetic reconnection are desired. In the MMS era, the advancement of simulations has also opened new possibilities for close comparisons between observations and simulations at different scales. Applying these simulations, which are “validated” by comparison with in-situ measurements, to other systems in different parameter regimes via next-generation computing techniques is expected to further advance our understanding of the physics of magnetic reconnection.
